# Out-of-hospital cardiac arrest event after cancer diagnosis: a korean metropolitan cohort study

**DOI:** 10.1186/s12885-025-13717-9

**Published:** 2025-02-19

**Authors:** Sun Young Lee, Jeong Ho Park, Yoonjic Kim, Jungah Lee, Young Sun Ro, Kyoung Jun Song, Sang Do Shin

**Affiliations:** 1 Public Healthcare Center, Seoul National University Hospital, Seoul, South Korea; 2 Department of Human Systems Medicine, Seoul National University College of Medicine, Seoul, South Korea; 3 Laboratory of Emergency Medical Services, Seoul National University Hospital Biomedical Research Institute, Seoul, South Korea; 4 Department of Emergency Medicine, Seoul National University College of Medicine and Hospital, Seoul, South Korea; 5 Disaster Medicine Research Center, Seoul National University Medical Research Center, Seoul, Republic of Korea, Seoul, South Korea; 6 Department of Emergency Medicine, Seoul National University Boramae Medical Center, Seoul, South Korea

**Keywords:** Out-of-hospital cardiac arrest, Cancer, Incidence

## Abstract

**Background:**

The importance of assessing out-of-hospital cardiac arrest (OHCA) risk in cancer patients is increasing as cancer incidence rises in aging populations.

**Objective:**

This study aimed to investigate the association between newly diagnosed cancer and OHCA risk using a metropolitan cohort from South Korea.

**Methods:**

A population-based retrospective cohort study was conducted, linking the nationwide OHCA registry with the National Health Information Database. The study included adults aged 40 years or older, residing in Seoul between 2015 and 2018, with no history of cancer or OHCA. The main exposure was cancer development. The primary outcome was the occurrence of OHCA with medical cause. Adjusted hazard ratios (aHRs) and 95% confidence intervals (CIs) were calculated using a cause-specific hazard model considering death as a competing risk. Analyses stratified by age group and cancer type were also conducted.

**Results:**

During a follow-up period of up to 4 years for 5,450,438 individuals, 174,785 participants developed cancer. The incidence rates of OHCA per 100,000 person-years were 54.0 in non-cancer and 145.0 in cancer groups, respectively. The aHR (95% CI) for OHCA associated with cancer development was 3.18 (2.97–3.41). The aHR (95% CI) for OHCA was highest in the 40–49 years of age group (7.52 [5.52–10.25]), followed by 50–59 years old (6.66 [5.56–7.97]) compared to older age groups. By cancer type, pancreatic, lung, biliary tract, and liver cancer were associated with a significantly increased risk of OHCA.

**Conclusion:**

We found an association between newly diagnosed cancer and the occurrence of OHCA. Our findings underscore the importance of tailored risk assessments and proactive care planning for patients with cancer.

## Key messeages

### What is already known on this topic

Previous studies have identified various risk factors for OHCA, but there is a growing need to assess these risks specifically in cancer patients due to the rising incidence of cancer and its complex relationship with cardiovascular disease.

### What this study adds

- This study demonstrates an association between newly diagnosed cancer and an increased risk of OHCA with medical cause, especially among younger patients.
- The study also highlights that the risk of OHCA varies significantly by cancer type, with pancreatic, lung, biliary tract, and liver cancers showing the highest risks.

### How this study might affect research, practice or policy

These findings emphasize the need for tailored risk assessments and proactive care planning for cancer patients to mitigate the risk of OHCA and ensure appropriate resuscitation efforts.

## Introduction

Out-of-hospital cardiac arrest (OHCA) is a critical global health issue, with an annual incidence of 40 to 100 per 100,000 individuals and survival rates below 10% in most countries [1]. Despite a growing body of research on OHCA risk factors, including cardiovascular comorbidities, socioeconomic status, and behavioral factors [2, 3], less attention has been paid to the occurrence of OHCA events in cancer patients. As cancer incidence and prevalence continue to rise, especially in aging populations [4], understanding the occurrence of OHCA events following a cancer diagnosis is becoming increasingly important.

Recent studies have suggested a complex relationship between cancer and cardiovascular events, underscoring the shared risk factors and pathophysiological mechanisms [5–7]. In particular, the fact that chronic diseases such as diabetes and the use of medications such as metformin affect both cancer and cardiovascular disease development also contributes to the complex relationship between cancer and cardiovascular events [8, 9]. However, there remains a gap in understanding how frequently OHCA events occur in patients with cancer, and this gap hinders proactive, informed decision-making regarding appropriate care when OHCA occurs in patients with cancer. Insufficient provision of necessary resuscitation and the administration of unnecessary resuscitation are both common issues in patients with cancer [10–12]. A better understanding the OHCA risk in these patients can support informed decision-making in advance.

This study aimed to investigate the incidence of OHCA with medical cause in patients with cancer using metropolitan cohort data from South Korea. We compared the occurrence of OHCA in patients with newly diagnosed cancer to those without cancer and explored variations by sex, age group, and cancer type.

## Methods

### Study design and setting

A population-based retrospective cohort study was conducted, utilizing a linkage database that connects the nationwide OHCA registry with the National Health Information Database (NHID) [13].

Korea has a population of 50 million across 17 provinces. Seoul, the capital of Korea, is a metropolitan city with a population of approximately 10 million. Korea has operated a national health insurance (NHI) system under a single payer since 1989, mandating enrollment for all citizens by law [13]. The NHI offers broad coverage for serious diseases such as cancer, resulting in reduced out-of-pocket expenses for cancer-related treatments [14]. The Korean prehospital Emergency Medical Service (EMS) system was exclusively operated by the National Fire Agency (NFA). EMS providers provide cardiopulmonary resuscitation (CPR) according to Korean CPR guidelines based on the American Heart Association guidelines [15, 16].

This study is an observational study conducted using an existing database and was carried out in accordance with the Strengthening the Reporting of Observational Studies in Epidemiology statement.

### Data sources

The NHID, a nationally representative database managed by the Korean NHI Corporation [13], encompasses demographic information and all healthcare records including diagnoses, procedures, and prescriptions for inpatient and outpatient visits [13, 17].

The Korean OHCA registry includes all EMS-assessed OHCA cases in Korea since 2006. The NFA and the Korean Disease Control and Prevention Agency (KDCA) cooperated to build a database by integrating three NFA databases (ambulance run sheets, the EMS cardiac arrest registry for the Utstein criteria, and the dispatch CPR registry) and the OHCA database with hospital treatment results. The KDCA manages the entire database and conducts a monthly quality management process in accordance with the Statistics Act. The NHID of the study participants and the nationwide OHCA database were linked using an individual’s unique resident registration number. Subsequently, the database was completed by deleting the resident registration number, which is a personal information. By linking two representative data sources, a cohort database covering Seoul City was established and used in the study.

### Study population

The study population comprised adults aged 40 or older who lived in Seoul from 2015 to 2018. Considering that the incidence of cancer and cardiac arrest generally increases with age, the study subjects were people over 40 years of age to avoid too rare exposure and outcome. To include participants with neither cancer nor OHCA, patients diagnosed with cancer in 2014 and OHCA survivors between 2011 and 2014 were excluded. Patients who experienced OHCA before cancer diagnosis were excluded to establish a temporal causal relationship between cancer development and OHCA occurrence.

### Outcome

The study outcome was the occurrence of OHCA with a medical cause extracted from a Korean OHCA registry. The etiology of cardiac arrest was identified by reviewing the medical records extracted by medical record reviewers from the KDCA. Patients with no cancer were observed from January 1, 2015, when the cohort was established, whereas patients with cancer were observed from the time of cancer diagnosis. The study participants were observed until the development of OHCA, death, or the end of the study period (December 31, 2018).

### Variables and measurements

The primary exposure is newly diagnosed cancer. Cancer was defined, based on previous studies [18, 19], as a patient who visited the outpatient clinic more than three times or was hospitalized more than once within a year with a cancer diagnosis (malignancy and neoplasm: C code) based on the International Classification of Diseases, 10th edition (ICD-10).

We collected the following variables from the NHID: 1) demographics (age, sex, and type of insurance [NHI or medical aid]), 2) comorbidities (diabetes, hypertension, dyslipidemia, and Charlson comorbidity index [CCI] score), and 3) cancer diagnosis (ICD-10 code and date of diagnosis). CCI includes myocardial infarction, congestive heart failure, peripheral vascular disease, cerebrovascular accident or transient ischemic attacks, dementia, chronic pulmonary disease, connective tissue disease, peptic ulcer disease, liver disease, diabetes mellitus, hemiplegia, chronic kidney disease, solid tumor, leukemia, lymphoma, and acquired immune deficiency syndrome. Comorbidities were evaluated based on the presence of the disease 1 year prior to the initiation of the cohort study on January 1, 2015. Following the definitions of previous studies using the NHID, hypertension and diabetes were defined as one or more visits (outpatient or hospitalization) with corresponding diagnosis codes (hypertension I10-13 and I15; diabetes E11-14) and prescribed medication with the corresponding diagnosis. Dyslipidemia was defined as one or more visits (outpatient or hospitalization) with a diagnosis of E78 [20]. CCI was defined based on the ICD-10 code in the NHID [21].

### Statistical analysis

The characteristics of the study populations with and without cancer were compared. The characteristics of the populations with and without OHCA among patients with cancer were also compared. Categorical variables are presented as numbers and proportions, and continuous variables as medians and interquartile ranges (IQR) or means and standard deviations (SD). Categorical variables were compared using the chi-square test and continuous variables were compared using the Wilcoxon rank-sum test or t-test, as appropriate.

Using a cause-specific hazard model, hazard ratios (HRs) with 95% confidence intervals (CIs) for OHCA occurrence were calculated, considering death as a competing risk, given the higher risk of death among patient with cancer compared to non-cancer population. The proportional hazard assumption was examined by plotting the log–log survival function, and no violations of this assumption were detected. Three models were used to adjust for potential confounding factors at baseline. Model 1 was adjusted for sex and age group (40–49, 50–59, 60–69, and > 70). Model 2 was further adjusted for the insurance type (NHI and medical aid). Model 3 was further adjusted for important comorbidities (diabetes, hypertension, and dyslipidemia), which are known cardiovascular risk factors and associated with cancer. A stratified analysis by sex and age group was conducted, and OHCA risk by cancer type (most common 10 types of cancers: stomach [C16], lung [C33-34], colon [C18-20], breast [C50], liver [C22], prostate [C61], pancreas [C25], biliary tract [C23-24], thyroid [C73], bladder [C67], and others) was also evaluated. Certain types of cancer, such as breast cancer (analyzed mainly in women) and prostate cancer (analyzed only in men), were analyzed for that specific sex only. The Kaplan–Meier curve was utilized to delineate the time to occurrence of OHCA, stratified by the presence or absence of cancer.

All statistical analyses were conducted using the SAS software (version 9.4; SAS Institute Inc., Cary, NC, USA). The *p*-values were based on a two-tailed significance level of 0.05.

### Ethics statement

The Institutional Review Board (IRB) of the study institution approved the study protocol (IRB number: E-2007–136-1143) and this study used NHIS-NHID (NHIS-2021–1-195) created by the National Health Insurance Service (NHIS). The requirement for informed consent was waived by the IRB because the data were analyzed retrospectively and anonymously.

## Results

### Characteristics of study participants by cancer

**Fig. 1:**
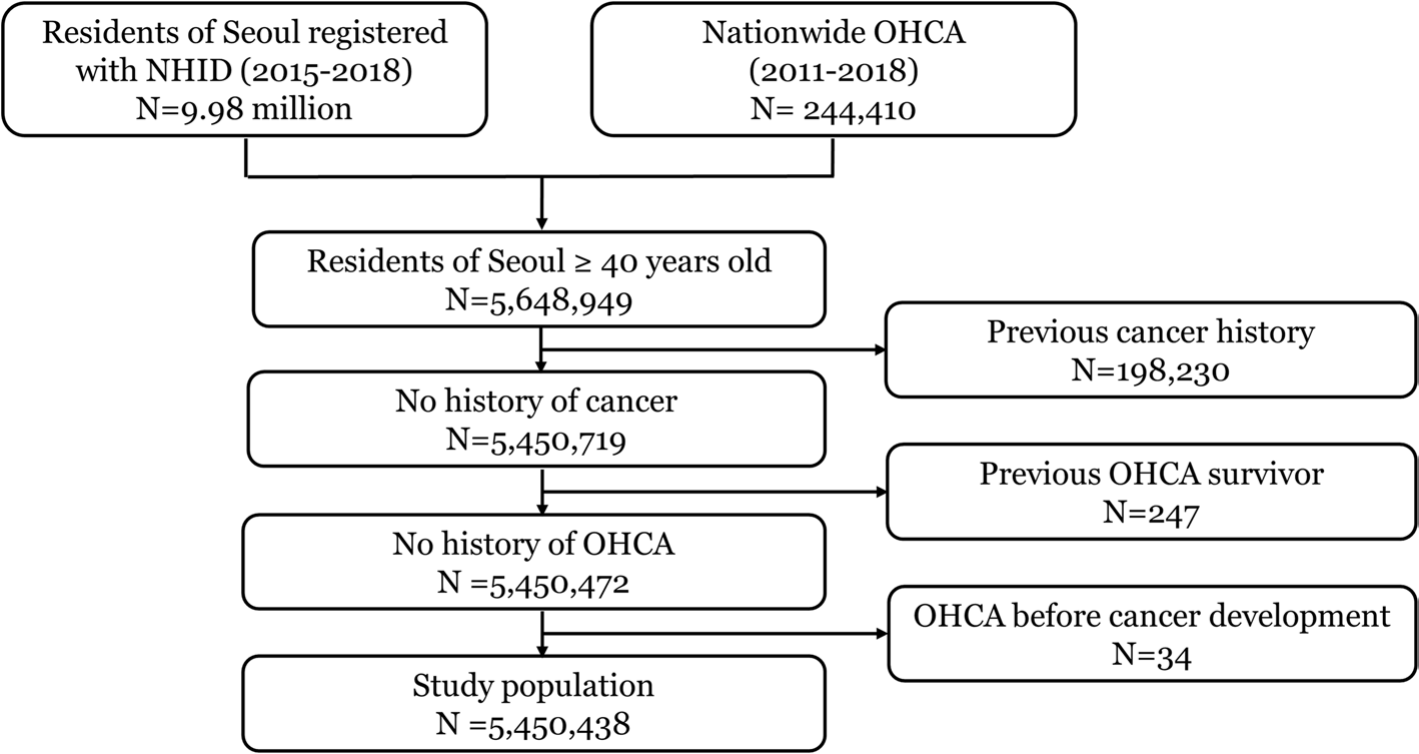
Patient flow. NHID, National Health Information Database; OHCA, out-of-hospital cardiac arrest

Among the 5,648,949 patients, we excluded 198,230 patients treated for cancer in 2014 prior to the study period, 247 OHCA survivors between 2011 and 2014, and 34 patients with OHCA before cancer diagnosis. The final study population consisted of 5,450,438 individuals (Fig. 1).

**Fig. 2:**
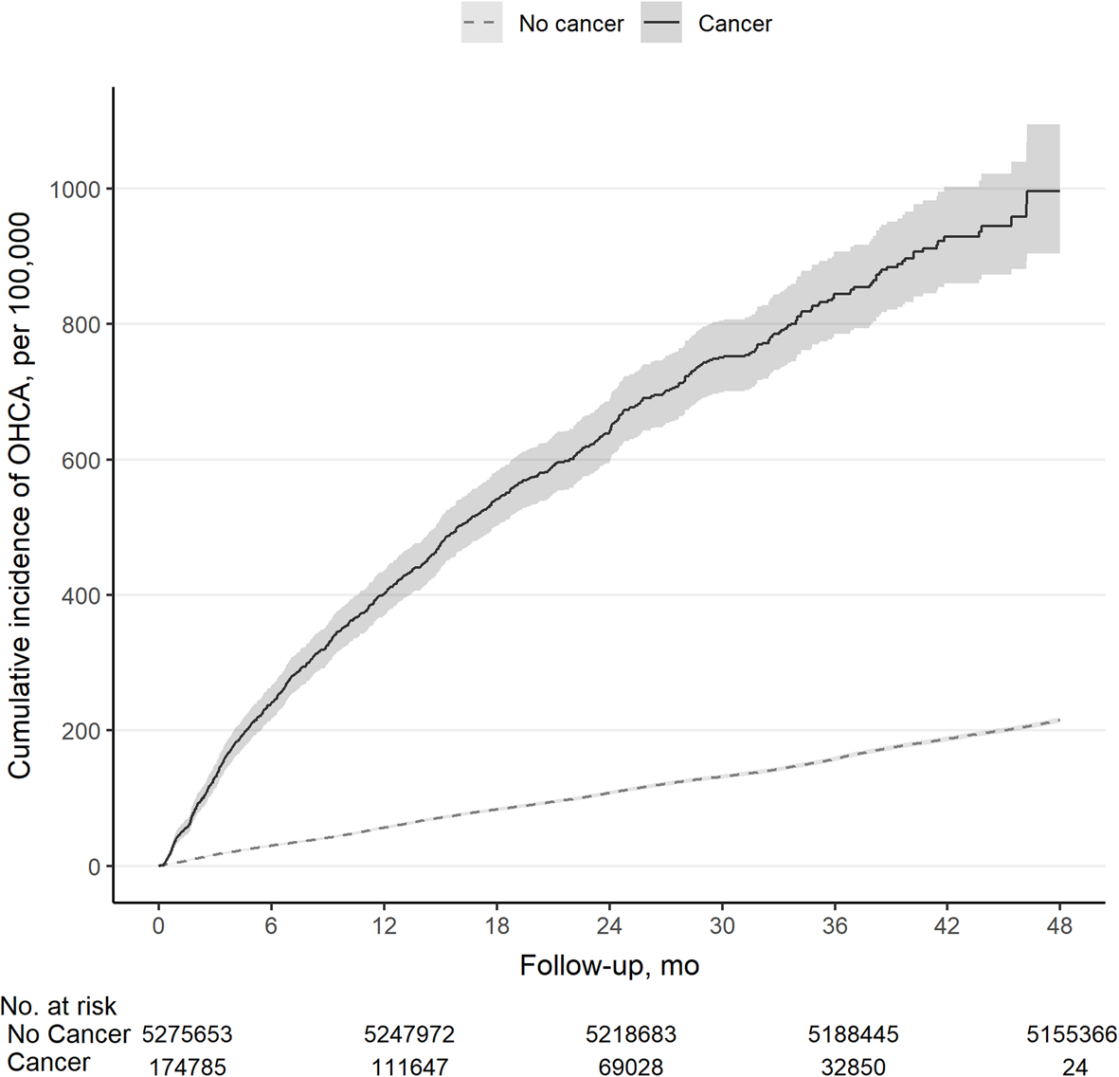
Cumulative incidence of out-of-hospital cardiac arrest by cancer status. OHCA, out-of-hospital cardiac arrest

During 21,151,497.6 person-years of follow-up (mean ± SD 46.6 ± 6.7 months) in the 5,450,438 individuals, 174,785 participants developed cancer, and the remaining 5,275,653 participants were included in the non-cancer group. Compared with participants who did not develop cancer, those who developed cancer were older (median [IQR], 63 [54–72] versus 54 [47–63]) and had more comorbidities (diabetes 14.9% vs. 8.9%, hypertension 37.3% vs. 24.7%, and dyslipidemia 35.4% vs. 26.4%, all *p* < 0.01). During the observation period (median 20.4 months in patients with cancer and 47.5 months in non-cancer group), OHCA occurred in 0.5% (929) in patients with cancer and 0.2% (11,257) in non-cancer group (Table 1) (Fig. 2).

**Table 1:** Characteristics of the study population by cancer development

		Total		Cancer		Non-cancer		*p* value
		N	%	N	%	N	%	
Total		5,450,438	100.0	174,785	3.2	5,275,653	96.8	
Sex	Female	2,826,413	51.9	83,721	47.9	2,742,692	52.0	< 0.01
Age group								< 0.01
	40–49	1,853,380	34.0	27,922	16.0	1,825,458	34.6	
	50–59	1,708,122	31.3	43,835	25.1	1,664,287	31.5	
	60–69	1,074,448	19.7	47,195	27.0	1,027,253	19.5	
	70 ~	814,488	14.9	55,833	31.9	758,655	14.4	
	Median (IQR)	55.0 (47.0–63.0)		63.0 (54.0–72.0)		54.0 (47.0–63.0)		< 0.01
Type of insurance								< 0.01
	NHI	5,299,823	97.2	167,548	95.9	5,132,275	97.3	
	Medical aid	150,615	2.8	7,237	4.1	143,378	2.7	
Comorbidities								
	Diabetes	493,628	9.1	26,106	14.9	467,522	8.9	< 0.01
	Hypertension	1,366,429	25.1	65,143	37.3	1,301,286	24.7	< 0.01
	Dyslipidemia	1,456,203	26.7	61,788	35.4	1,394,415	26.4	< 0.01
CCI score								< 0.01
	0	2,897,395	53.2	72,137	41.3	2,825,258	53.6	
	1	1,309,998	24.0	44,054	25.2	1,265,944	24.0	
	2	644,903	11.8	26,544	15.2	618,359	11.7	
	3	308,213	5.7	14,911	8.5	293,302	5.6	
	≥ 4	289,929	5.3	17,139	9.8	272,790	5.2	
Follow-up period, month								< 0.01
	Mean +—SD	46.6 +—6.7		20.4 +—14.2		47.5 +—4.1		
OHCA		12,186	0.2	929	0.5	11,257	0.2	< 0.01

*IQR* interquartile range, *NHI* National Health Insurance, *CCI* Charlson Comorbidity Score, *SD* standard deviation, *OHCA* out-of-hospital cardiac arrest

Of the 174,785 patients with cancer, those who experienced OHCA were older (median [IQR], 72 [63–78] versus 63 [54–72]) and had more comorbidities (diabetes 23.5% vs. 14.9%, hypertension 47.3% vs. 37.2%, and dyslipidemia 37.2% vs. 35.3%, all *p* value < 0.01) than those who did not develop OHCA (Table 2).

**Table 2:** Characteristics of patients with cancer by out-of-hospital cardiac arrest

		Total		OHCA		non-OHCA		*p* value
		N	%	N	%	N	%	
Total		174,785	100.0	929	100.0	173,856	100.0	
Sex	Female	83,721	47.9	251	27.0	83,470	48.0	< 0.01
Age group								< 0.01
	40–49	27,922	16.0	43	4.6	27,879	16.0	
	50–59	43,835	25.1	132	14.2	43,703	25.1	
	60–69	47,195	27.0	215	23.1	46,980	27.0	
	70 ~	55,833	31.9	539	58.0	55,294	31.8	
	Median (IQR)	63.0 (54.0–72.0)		72.0 (63.0–78.0)		63.0 (54.0–72.0)		< 0.01
Type of insurance								0.11
	NHI	167,548	95.9	881	94.8	166,667	95.9	
	Medical aid	7,237	4.1	48	5.2	7,189	4.1	
Comorbidities								
	Diabetes	26,106	14.9	218	23.5	25,888	14.9	< 0.01
	Hypertension	65,143	37.3	439	47.3	64,704	37.2	< 0.01
	Dyslipidemia	61,788	35.4	346	37.2	6,1442	35.3	< 0.01
CCI score								< 0.01
	0	72,137	41.3	279	30.0	71,858	41.3	
	1	44,054	25.2	221	23.8	43,833	25.2	
	2	26,544	15.2	153	16.5	26,391	15.2	
	3	14,911	8.5	107	11.5	14,804	8.5	
	≥ 4	17,139	9.8	169	18.2	16,970	9.8	
Follow-up period, month								< 0.01
	Mean +—SD	20.4 +—14.2		11.4 +—10.1		20.4 +—14.2		

*IQR* interquartile range, *NHI* National Health Insurance, *CCI* Charlson Comorbidity Score, *SD* standard deviation, *OHCA* out-of-hospital cardiac arrest

### HRs for OHCA associated with cancer by age and sex

During the observation period, the OHCA incidence rates per 100,000 person-years were 54.0 in the non-cancer group and 145.0 in patients with cancer, respectively. After adjusting for age, sex, health insurance type, and comorbidities, the adjusted HR (95% CIs) for OHCA associated with cancer development was 3.18 (2.97–3.41). By sex group, the adjusted HR for OHCA was 3.32 (3.06–3.60) in the male group and 3.20 (2.81–3.64) in the female group. By age group, the adjusted HR for OHCA was highest in the 40–49-year-old group (7.52 [5.52–10.25]), followed by the 50–59-year-old group (6.66 [5.56–7.97]) and the 60–69-year-old group (4.58 [3.97–5.29]) The *p*-value in all logistic regression model was < 0.01. (Table 3).

**Table 3:** Hazard ratios for out-of-hospital cardiac arrest associated with cancer development by sex and age

		Exposure	Person-years	Event	OHCA incidence rate^*^	Crude			Model 1^a^			Model 2^b^			Model 3^c^		
				N		HR	95% CI		aHR	95% CI		aHR	95% CI		aHR	95% CI	
Total		Non-cancer	20,855,192.6	11,257	54.0	1.00			1.00			1.00			1.00		
		Cancer	296,305.0	929	313.5	5.70	5.33	6.10	3.21	3.00	3.44	3.21	3.00	3.44	3.18	2.97	3.41
Sex	Male	Non-cancer	10,005,145.9	6,954	69.5	1.00			1.00			1.00			1.00		
		Cancer	146,840.7	678	461.7	6.53	6.03	7.08	3.37	3.11	3.66	3.37	3.10	3.65	3.32	3.06	3.60
	Female	Non-cancer	1,085,0046.7	4,303	39.7	1.00			1.00			1.00			1.00		
		Cancer	149,464.3	251	167.9	4.15	3.65	4.72	3.24	2.84	3.68	3.23	2.84	3.67	3.20	2.81	3.64
Age	40 ~ 49	Non-cancer	7,283,503.7	958	13.2	1.00			1.00			1.00			1.00		
		Cancer	51,608.9	43	83.3	6.39	4.69	8.69	8.13	5.97	11.08	7.96	5.84	10.84	7.52	5.52	10.25
	50 ~ 59	Non-cancer	6,629,552.3	1,728	26.1	1.00			1.00			1.00			1.00		
		Cancer	79,193.7	132	166.7	6.49	5.42	7.76	6.97	5.82	8.34	6.90	5.76	8.25	6.66	5.56	7.97
	60 ~ 69	Non-cancer	4,078,322.8	2,045	50.1	1.00			1.00			1.00			1.00		
		Cancer	82,778.6	215	259.7	5.22	4.52	6.02	4.71	4.08	5.44	4.68	4.06	5.40	4.58	3.97	5.29
	70 ~	Non-cancer	2,863,813.7	6,526	227.9	1.00			1.00			1.00			1.00		
		Cancer	82,723.7	539	651.6	2.79	2.55	3.05	2.52	2.30	2.76	2.52	2.30	2.76	2.51	2.30	2.75

*N* number, *OHCA* out-of-hospital cardiac arrest, *HR* hazard ratio, *CI* confidence interval, *aHR* adjusted HR

^*^per 100,000 person-years

^a^Model 1 adjusted for age (10 years old group) and sex

^b^Model 2 adjusted for Model 1 and type of insurance

^c^Model 3 adjusted for Model 2 and comorbidities (diabetes, hypertension, and dyslipidaemia)

### HRs for OHCA associated with cancer by type of cancer

By type of cancer, pancreas (HR [95% CI] 7.59 [5.64–10.22]), lung (HR [95% CI] 7.29 [6.36–8.35]), biliary tract (HR [95% CI] 6.18 [4.58–8.34]), and liver (HR [95% CI] 5.86 [4.79–7.16]) cancer were associated with a significantly increased risk of OHCA (Table 4).

**Table 4:** Hazard ratios for out-of-hospital cardiac arrest associated with cancer development by type of cancer

Exposure		Person-years	Event	OHCA incidence rate^*^	Crude			Model 1			Model 2			Model 3			
			N		HR	95% CI		aHR	95% CI		aHR		95% CI	aHR	95% CI		*P*-value
Non-cancer		20,855,192.6	11,257	54.0	1.00			1.00			1.00			1.00			
Cancer																	
1	Stomach	38,464.1	94	244.4	4.47	3.65	5.47	2.10	1.72	2.58	2.11	1.72	2.59	2.09	1.71	2.56	< 0.01
2	Lung	22,383.8	215	960.5	17.27	15.08	19.78	7.36	6.42	8.44	7.32	6.39	8.39	7.29	6.36	8.35	< 0.01
3	Colon	42,188.3	119	282.1	5.17	4.31	6.19	2.48	2.06	2.97	2.47	2.06	2.96	2.40	2.01	2.88	< 0.01
4	Breast	39,803.4	11	27.6	0.51	0.28	0.92	0.88	0.49	1.60	0.89	0.49	1.61	0.89	0.49	1.60	0.691
5	Liver	15,597.9	97	621.9	11.23	9.19	13.72	6.35	5.20	7.77	6.28	5.14	7.68	5.86	4.79	7.16	< 0.01
6	Prostate	20,773.9	40	192.5	3.52	2.58	4.81	0.96	0.71	1.31	0.97	0.71	1.33	0.99	0.73	1.36	0.968
7	Pancreas	4,970.1	44	885.3	15.67	11.65	21.08	7.91	5.88	10.64	7.92	5.89	10.66	7.59	5.64	10.22	< 0.01
8	Biliary tract	5,092.4	43	844.4	15.16	11.23	20.45	6.29	4.66	8.49	6.30	4.67	8.51	6.18	4.58	8.34	< 0.01
9	Thyroid	32,806.7	8	24.4	0.45	0.22	0.90	0.73	0.36	1.46	0.73	0.37	1.47	0.74	0.37	1.48	0.398
10	Bladder	9,427.0	33	350.1	6.40	4.55	9.01	2.23	1.59	3.14	2.21	1.57	3.12	2.18	1.55	3.08	< 0.01
	Others	64,797.5	225	347.2	6.33	5.54	7.23	3.86	3.38	4.40	3.85	3.37	4.40	3.83	3.35	4.37	< 0.01

*N* number, *OHCA* out-of-hospital cardiac arrest, *HR* hazard ratio, *CI* confidence interval, *aHR* adjusted HR

^*^per 100,000 person-years

^a^Model 1 adjusted for age (10 years old group) and sex

^b^Model 2 adjusted for Model 1 and type of insurance

^c^Model 3 adjusted for Model 2 and comorbidities (diabetes, hypertension, and dyslipidaemia)

## Discussion

In this study, using a cohort dataset from a metropolitan city, we observed that cancer development was associated with the occurrence of OHCA, with the risk varying by age. Younger individuals exhibited a relatively greater risk of OHCA in the presence of cancer compared to older adults, whereas no significant differences were observed based on sex. Substantial variability in OHCA risk has been noted across different types of cancer, with pancreatic, lung, biliary tract, and liver cancers demonstrating significant increases in OHCA risk.

We identified an increase in the occurrence of OHCA from the time of cancer diagnosis compared to that in the non-cancer group. Patients with cancer had a higher risk of OHCA than those without. Despite being older and having more known cardiovascular disease risk factors, such as diabetes and hypertension, than non-cancer patients (Table 1), the risk of OHCA remained elevated after adjusting for these factors. As an observational study, identifying the specific mechanisms contributing to the higher OHCA risk in patients with cancer is challenging. However, this increased risk may be attributed to both the cancer itself and the adverse cardiac effects caused by anti-cancer therapy [22–24]. Previous research has reported that many cancers elevate the risk of coronary heart disease (CHD) within 6 months of diagnosis, necessitating more aggressive management for classical CHD risk factors [23, 25]. Furthermore, targeted cancer therapy and immunotherapy, along with conventional chemotherapy and radiation therapy, are associated with adverse cardiac effects [24, 26], including arrhythmias, cardiomyopathy, and increased risk of arterial vascular disease or venous thromboembolism [24]. In addition, OHCA can encompass both an acute cardiac event that requires immediate treatment, as well as a natural process of death in patients nearing the end of life and for whom such an event may occur without prior preparation. In patients with cancer, this end-of-life process is likely to occur at a higher rate compared to those without cancer.

When analyzing the risk of OHCA based on age and sex, there was no significant difference by sex; however, there was a notable variation in risk across age groups. The incidence of OHCA increased with age, and in younger age groups, the elevated risk in patients with cancer was relatively more pronounced (Table 3). Since OHCA is frequent in the elderly, even without cancer, the contribution of cancer to OHCA risk may be relatively small [27]. This aligns with previous studies indicating that risk factors associated with OHCA, such as pre-arrest comorbidity burden and socioeconomic position, have a greater impact in younger age groups with a lower baseline risk of cardiac arrest [28]. In the younger age group, where the overall incidence of OHCA is exceptionally low in non-cancer patients, the relative risk of OHCA in patients with cancer may be heightened [29]. For individuals newly diagnosed with cancer, it is vital to assess the risk of cardiovascular disease, including OHCA, and to screen for and control cardiovascular disease risk factors, especially in younger patients [30].

We evaluated the risk of OHCA according to cancer type and found considerable variation in risk by type. Some types of cancer increase the risk of OHCA by more than seven times compared to individuals without cancer. This discrepancy may be attributed to variations in mortality, complications, and treatment methods depending on the type of cancer [31]. Cancers with a high mortality and complications, such as lung and pancreatic cancer, significantly elevate the risk of OHCA [31]. High mortality from cancer and complications of chemotherapy specific to each cancer type may influence the risk of OHCA [24, 32]. Certain drugs with known cardiotoxicity, such as 5-fluorouracil or capecitabine, and the risks of new drugs, such as targeted cancer therapy and immunotherapy, have been reported [24, 32]. It is also necessary to prepare for the occurrence of OHCA in patients with cancer using drugs associated with adverse cardiac effects. On the other hand, during the observation period, some types of cancer showed a similar risk of OHCA as non-cancer patients. The 5-year relative survival rate of thyroid cancer and the 10-year survival rate of prostate cancer are approximately 98%, respectively and breast cancer has a 5-year relative survival rate of 91% even in invasive cases [31]. OHCA may not have occurred in specific types of cancer owing to the insufficient observation period for slow-growing cancers. The limited observation period of 4 years in this study may have contributed to these results by not capturing the occurrence of OHCA in slow growing cancer. Additionally, compared with individuals without cancer, those diagnosed with cancer diligently visit the hospital and take good care of their health after diagnosis, resulting in fewer health problems in the early stages of cancer development.

The prevalence of cancer is increasing, and studies on OHCA in patients with cancer are also increasing. Previously, resuscitation of patients with cancer in OHCA was considered futile; however, recent studies indicate that even patients with cancer can improve outcomes through active treatments such as extracorporeal CPR, considering specific patient conditions [33, 34]. In patients with cancer, a plan for unexpected events in the course of treatment, including OHCA, should be developed in advance so that necessary treatment can be employed. For vulnerable populations at high risk of OHCA, it may also be helpful to educate those close to them, such as family members, to recognize the occurrence of cardiac arrest and provide bystander CPR quickly. Physicians should be aware of the risk of cardiac arrest, actively manage cardiovascular risk factors, and monitor patients to respond appropriately, especially for certain types of cancer and young patients with cancer at a high risk of OHCA. Additionally, timely advanced care planning is necessary for patients with cancer who are expected to die to avoid OHCA and unnecessary resuscitation efforts. While the patient is at home, cardiac arrest that occurs as the dying period progresses may be reported to EMS, potentially resulting in an ambulance being dispatched and unnecessary resuscitation. This can be an unnecessary use of EMS resources and can be traumatic for the patient and their family. Terminal stage cancer patients who are expected to die require appropriate guidance and education to ensure suitable end-of-life care.

### Limitations

This study has limitations. First, the accuracy of the cancer diagnosis cannot be measured directly. However, additional insurance benefits are granted when patients are registered with cancer at National Cancer Registry and use healthcare services for cancer-related problems, making it advantageous for the cancer diagnosis to be included in the NHID for insurance benefits [14]. Physicians carefully input a cancer diagnosis at each visit for patients with cancer, reducing the likelihood of a cancer diagnosis being included in the claim data of non-cancer patients. Second, the cancer stage and treatment status were not analyzed in this study due to limitations in the available data. The risk of cardiac arrest may vary depending on the stage of cancer. Also, different types of treatment, such as chemotherapy drug, may pose different risks. Third, this study was followed up for a maximum of 4 years, which is relatively short considering the high survival rate and long disease duration in patients with some type of cancer. In particular, the observation period. The observation period may have been too short to observe the risk of cardiac arrest in cancer with slow progression and high 5-year survival rate. More extensive follow-up studies are required to gain a comprehensive understanding of OHCA occurrence in patients with cancer. Fourth, as patients with cancer are frequently hospitalized, cases of in-hospital cardiac arrest were excluded from the analysis. Additionally, cases that did not receive resuscitation because death was certain at the scene such as post-mortem rigor, were excluded. Fifth, as a retrospective observational study, the analysis took confounders into consideration, but it was not possible to consider all possible confounders that could affect the outcome. There will be confounder effects that are not adjusted for. Lifestyle factors such as smoking and alcohol consumption, which affect both cancer and cardiac arrest, and specific factors such as obesity indices assessed by body mass index and metformin use in diabetes, may have affected the study results. Further research is needed into this in the future. This exclusion may have contributed to an underestimation of the impact of cancer on cardiac arrest. Finally, this study was conducted on the population of a metropolitan city where the NHI was applied, making it difficult to generalize the findings to other populations with different races and healthcare environments.

## Conclusions

In conclusion, this retrospective cohort study demonstrated an association between cancer diagnosis and an increased risk of OHCA. Compared to the non-cancer group, the risk of OHCA remained elevated in patients with cancer after adjusting for known cardiovascular risk factors, with younger patients showing a particularly pronounced relative risk. Considering that OHCA can occur both as an unexpected cardiovascular event requiring preparation and treatment, and as part of the natural course of death in patients with terminal-stage cancer, tailored risk assessment and proactive approaches for advanced care planning are necessary for newly diagnosed cancer patients. Such an approach would help to optimizing the care and outcomes of patients with cancer, particularly given the increasing prevalence of cancer and improved survival rates.

## Data Availability

The data cannot be shared at this time, as access is restricted by the National Health Insurance Service and the Korea Disease Control and Prevention Agency. These organizations permit data sharing only to researchers who have obtained prior approval. Permission to access the dataset can be requested by contacting the relevant authority via email (kjisu0111@korea.kr).

## Abbreviations

CCI: Charlson comorbidity index
CHD: Coronary heart disease
CI: Confidence interval
CPR: Cardiopulmonary resuscitation
EMS: Emergency medical service
ICD-10: International classification of disases, 10th edition
IQR: Interquartile range
KDCA: Korean disease control and prevention agency
NCR: National cancer registry
NFA: National fire agency
NHI: National health insurance
NHID: National health information database
OHCA: Out-of-hospital cardiac arrest
SD: Standard deviation
HR: Hazard ratio
